# Naturally Occurring PCSK9 Inhibitors

**DOI:** 10.3390/nu12051440

**Published:** 2020-05-16

**Authors:** Maria Pia Adorni, Francesca Zimetti, Maria Giovanna Lupo, Massimiliano Ruscica, Nicola Ferri

**Affiliations:** 1 Department of Medicine and Surgery-Unit of Neurosciences, University of Parma, 43125 Parma, Italy; mariapia.adorni@unipr.it; 2 Dipartimento di Scienze degli Alimenti e del Farmaco, Università di Parma, 43124 Parma, Italy; francesca.zimetti@unipr.it; 3 Dipartimento di Scienze del Farmaco, Università degli Studi di Padova, 35121 Padova, Italy; mariagiovanna.lupo@phd.unipd.it; 4 Dipartimento di Scienze Farmacologiche e Biomolecolari, Università degli Studi di Milano, 20122 Milan, Italy; massimiliano.ruscica@unimi.it

**Keywords:** nutraceuticals, PCSK9, SREBP, HNF1α, berberine, cholesterol

## Abstract

Genetic, epidemiological and pharmacological data have led to the conclusion that antagonizing or inhibiting Proprotein convertase subtilisin/kexin type 9 (PCSK9) reduces cardiovascular events. This clinical outcome is mainly related to the pivotal role of PCSK9 in controlling low-density lipoprotein (LDL) cholesterol levels. The absence of oral and affordable anti-PCSK9 medications has limited the beneficial effects of this new therapeutic option. A possible breakthrough in this field may come from the discovery of new naturally occurring PCSK9 inhibitors as a starting point for the development of oral, small molecules, to be used in combination with statins in order to increase the percentage of patients reaching their LDL-cholesterol target levels. In the present review, we have summarized the current knowledge on natural compounds or extracts that have shown an inhibitory effect on PCSK9, either in experimental or clinical settings. When available, the pharmacodynamic and pharmacokinetic profiles of the listed compounds are described.

## 1. Introduction

Proprotein convertase subtilisin/kexin type 9 (PCSK9) is a pivotal regulator of low-density lipoprotein (LDL) receptor, and thus of LDL-cholesterol levels [1]. PCSK9 is mainly synthesized by the hepatocytes, where it undergoes an autocatalytic cleavage in the endoplasmic reticulum (ER) that allows the release of the mature PCSK9 from the endoplasmic reticulum (ER) to the Golgi [2,3,4,5]. PCSK9 is one of the 33 genes regulated by the sterol regulatory element (SRE) binding protein (SREBP) family of transcription factors [6]. When cell cholesterol depletion or inhibition of intracellular synthesis occurs, PCSK9 promoter activity is raised, leading to an increased transcription [7]. A second transcription factor involved in regulation of PCSK9 is the hepatocyte nuclear factor 1α (HNF1α) [8,9]. Once secreted, PCSK9 binds the epidermal growth factor-like repeat homology domain A (EGFA-like) of the LDL receptor (LDLR) through its catalytic domain. This phenomenon fosters the degradation of LDLR in lysosomes, instead of allowing it to recycle on the cell surface. This degrading activity reduces the number of LDLR on hepatocytes, and thus the uptake of circulating LDL particles by the liver. For this reason, PCSK9 genetic gain-of-function (GOF) mutations are associated to hypercholesterolemic conditions, and its pharmacological inhibition has been considered as a new line of intervention for preventing cardiovascular diseases [10,11,12].

At least two strategies have been developed to reduce PCSK9 plasma levels or to inhibit its binding to the LDLR, i.e., monoclonal antibodies and antisense oligonucleotides [5]. However, an optimal pharmacological strategy to inhibit PCSK9 may involve the identification and development of orally absorbed small molecules with anti-PCSK9 activity. The history of pharmacology has provided compelling evidence of the importance of identifying naturally occurring chemical entities with potential therapeutic activities. For this reason, in the present review, we summarized the current knowledge on natural compounds or extracts that have shown significant PCSK9 inhibitory activity.

## 2. Berberine

Plants belonging to the genus *Berberis* (Family: *Berberidaceae*) are widely distributed worldwide, with nearly 550 species. Several studies have reported traditional uses *Berberis* for the treatment of metabolic diseases (e.g., diabetes and hyperlipidemia). Various bioactive compounds, such as alkaloids, polyphenols, flavonoids, anthocyanins, etc., have been found in *Berberis* species. 

Berberine, originally isolated from *Huanglian* (*Coptis chinensis, Franch. ranunculaceae*), is a quaternary ammonium salt belonging to a group of benzylisoquinoline alkaloids (Table 1). The chemical name of berberine is 5,6-dihydro-9,10-dimethoxybenzo[g]-1,3-benzodioxolo[5,6-α]quinolizinium. *Berberine* is the most active compound reported from *Berberis* species, and it is considered to be highly effective against diabetes and other metabolic diseases [13,14,15]. Berberine is present in roots, rhizomes, and stem bark of *Berberis*, and in other species of flowering plants *like Coptis rhizomes* and *Hydrastis Canadensis* [16].

The mechanism of action of the lipid-lowering effect of berberine was identified by screening 700 Chinese herbs with potential induction effect on LDLR expression [17]. Among different compounds tested, berberine showed the highest activity in increasing LDLR expression, suggesting a mechanism similar to hydroxymethylglutaryl-coenzyme A (HMG-CoA) reductase inhibitors, statins. However, berberine increases messenger ribonucleic acid (mRNA) and protein, as well as the function of hepatic LDLR, independently from the intracellular cholesterol levels. Thus, the upregulation of the LDLR, that is mediated by the activation of the transcription factor sterol regulatory element binding proteins (SREBPs) [18], is not involved in the action of berberine. Further investigation of the biological action of berberine led to the discovery that this natural compound prolongs the mRNA stability of LDLR approximately threefold (from 64 to 198 min).

After the discovery of the role of PCSK9 on LDLR, experimental studies were carried out in order to investigate if PCSK9 was involved in the mechanism of action of berberine. As previously described, the gene transcription of PCSK9 is mainly regulated by SREBP. However, key SRE motifs are usually adjacent to Sp1 (specific protein 1) or NF-Y (nuclear transcription factor Y) binding sites, and SREBPs work in concert with these coactivators to induce full transactivation. In this regard, the PCSK9 promoter has a unique sequence, with an HNF1 binding site, adjacent to SRE, as a critical regulatory sequence motif. HNF1α is, indeed, the predominant working partner for SREBP2 in the regulation of PCSK9 gene.

Starting from these relevant structural differences between the promoters of LDLR and PCSK9, berberine was shown to strongly reduce the PCSK9 mRNA levels in a time- and concentration-dependent manner [19]. This inhibitory effect is also independent from the SREBP pathway but related to HNF1α [9]. More interestingly, berberine inhibits PCSK9 protein expression and counteracts the inducing effect of various statins [9]. Indeed, berberine significantly reduced the expression of HNF1α (−60%), and only slightly of SREBP2 [9]. This effect is sufficient to block PCSK9 transcription without affecting LDLR expression. The synergy between SREBP2 and HNF1α is beneficial for LDLR expression, because SREBP2 is absolutely required for LDLR transcription. The fact that berberine increases LDLR protein level, both in vitro and in vivo [17,20,21], suggests that the balanced effects are in favor of LDLR mRNA stability. A more detailed study was conducted to investigate the mechanism underlying the inhibitory effect of berberine on HNF1α-mediated PCSK9 transcription. By using the proteasome inhibitor bortezomib, Dong et al. demonstrated that berberine accelerates the degradation of HNF1α by proteasome pathway. Thus, by blocking proteasome, the effect of berberine is antagonized, determining an increase of PCSK9 levels and a reduction of LDLR expression [22].

Finally, results from hamster experiments suggest that the effect of berberine on LDLR and plasma cholesterol is mainly derived from a systemic action, rather than an inhibition of gastrointestinal cholesterol absorption. These conclusions derive from the observation that intraperitoneal administration of berberine (20 mg/kg) has a stronger lipid-lowering effect than oral administration (100 mg/kg), and that oral berberine did not increase fecal lipids [21]. These results are particularly important considering that oral bioavailability of berberine is estimated to be around 0.37% [23]. In humans, the maximum concentration (Cmax) of berberine in plasma was measured at 0.4 ng/mL, after a single oral dose of 400 mg [24]. Intestinal first-pass elimination of berberine is considered the major barrier of its oral bioavailability, and that its high extraction and distribution in the liver could be other important factors that lead to its low plasma levels in rats. After intragastric dosing, berberine is widely distributed into various tissues, including liver, heart, kidney, spleen, lung, and even brain, with the liver being the most predominant organ, in which the mean level of berberine was approximately 70-fold greater than that in plasma [23].

Beyond the unfavorable physicochemical properties, a second factor that may negatively impact on the oral bioavailability of berberine is the fact that this compound is a substrate of some membrane transporters, including the P-glycoprotein (P-gp) and multidrug resistance protein 1 (MRP1) [25]. These transporters may limit berberine absorption by extruding it from the enterocytes.

At least four metabolites of berberine have been identified. Berberine phase I metabolites M1 via demethylation, M2 via demethylenation, and M3 (jatrorrhizine), from which derive phase II metabolites that are the corresponding glucuronide conjugates of M1, M2, and M3, respectively [23]. The unconjugated metabolites are the major forms present in tissues, including liver, heart, and kidney; however, glucuronides of phase I metabolites of berberine were the major forms in plasma after oral intake.

### 2.1. In Vitro Studies

The in vitro model utilized to predict the hypocholesterolemic action of nutraceuticals mainly involve the use of hepatoma cell line HepG2 or Huh7. The first report showing the effect of berberine on PCSK9 demonstrated that berberine, at concentration of 15 µg/mL (44 µM), reduced the amount of PCSK9 mRNA by 77%. Time course experiments demonstrated that berberine reduced PCSK9 mRNA within 8 h of incubation and reached a significant reduction at 12 h and 24 h (65% and 61%, respectively). The amount of PCSK9 secreted into the media of HepG2 cells treated with 15 µg/mL was reduced by 87% [19]. Under the same experimental condition, berberine increased the LDLR mRNA expression after 12 h and 24 h 1.9-fold and 2.1-fold, respectively [19]. Very similar results were observed by Li et al., with a significant reduction of PCSK9 levels at 12 h (−30%) in berberine-treated cells down to 23% after 48 h at the concentration of 20 µM (6.7 µg/mL) [9]. These studies also confirmed the antagonist effect of berberine on statin-induced PCSK9 mRNA levels [19].

When the analysis was extended to other genes involved in cholesterol homeostasis, berberine was shown to reduce the level of HMG-CoA reductase mRNA by 39%, without any significant effect on farnesyl-diphosphate synthase (FDPS) and 7-dehydrocholesterol reductase (DHCR7) mRNA, two enzymes involved in the synthesis of cholesterol. The same analysis conducted on non-SRE containing genes involved in lipid metabolism demonstrated that berberine increased the amounts of peroxisome proliferator-activated receptors alpha (PPARα) mRNA by 39% (*p* < 0.05), and SREBP2 mRNA by 74% (*p* < 0.05). These data demonstrated that there are no consistent effects of berberine on mRNA expression of genes with or without an SRE. Thus, berberine-mediated reduction in PCSK9 mRNA level does not involve the SREBP pathway. In addition, by using actinomycin D, berberine was shown to not alter the mRNA stability of PCSK9 while reducing its promoter activity [19].

Berberine metabolites can exert an extracellular signal-regulated kinase (ERK)-dependent PCSK9-lowering action, with berberrubine (M1) and its analogs being the most powerful [26].

### 2.2. In Vivo Studies

The first in vivo evidence of a lipid-lowering effect by berberine was reported in 2004 in hamsters fed high-fat and high-cholesterol diet (10% lard, 10% egg yolk powder and 1% cholesterol) [17]. This animal model was chosen since the kinetics of hepatic LDLR-mediated LDL clearance have been well characterized [27]. Treatment of these hyperlipidemic animals with berberine determined a time and dose-dependent reduction of total and LDL-cholesterol levels. According to the LDL kinetics, the effect on LDL-cholesterol was observed after 7 days of treatment, and at day 10 berberine reduced LDL-cholesterol by 26% and 42%, at a dose of 50 and 100 mg/kg/d, respectively. This effect was associated with increased LDLR mRNA (3.5-fold) and protein (2.6-fold) expressions in the liver [17]. However, the first in vivo report on the effect of berberine on PCSK9 derives from the analysis conducted in dyslipidemic C57BL/6 mice, in response to LPS-induced inflammation [28]. Berberine was given by oral gavage at the dose of 10 or 30 mg/kg per day and showed a significant and dose-dependent reduction of PCSK9 mRNA levels, induced by LPS, in the liver. This effect was associated with a significant increase of the LDLR mRNA [28]. Thus, although the animal model utilized cannot be consider optimal for studying the lipid-lowering properties of new agents, the data confirmed the in vitro analysis and reinforced the concept that berberine reduces PCSK9 transcription.

In contrast, different results were reported in a second study conducted in rats fed a high-fat diet (47% calories from fat, 20% calories from protein, 33% calories from carbohydrate) for 6 weeks [29]. 400 mg/kg/day of oral berberine significantly reduced LDL-cholesterol (−45%) and increased high-density lipoprotein (HDL) cholesterol (+45%), resulting in unchanged total cholesterol (TC) levels. Surprisingly, in response to high-fat diet, a significant increase of plasma levels of PCSK9 was observed, values that were further augmented in response to berberine (almost twofold higher) [29]. Similar trend was observed with simvastatin, utilized as control treated group.

To further investigate the effect of berberine on PCSK9, a third study was conducted in a similar model of hypercholesterolemic rats [30]. Rats were fed a high-fat diet (20% lard, 5% egg yolk powder, 2% cholesterol, 0.3% bile salts, and 0.2% Prothiucil) for 4 weeks, and then treated with berberine, at the dose of 156 mg/kg/day, by oral gavage once a day for 8 weeks. Berberine reduced TC, triglycerides (TG) and LDL-cholesterol by 68%, 66% and 83%, respectively. Interestingly, a berberine derivative, 8-hydroxydihydroberberine, considered to have a higher bioavailability than berberine, produced the same lipid-lowering effect when used at one fourth of the dose of berberine [30]. In this experimental model, a significant reduction of PCSK9 in the liver was found in berberine-treated animals compared to hypercholesterolemic controls [30].

Thus, it is possible to conclude that the animal models utilized had contrasting results, and are potentially not predictive of the human situation, where substantial differences on lipid metabolism are recognized.

### 2.3. Cilinical Studies

The first study that evaluated the effect of berberine in a Chinese population of hypercholesterolemic patients reported a significant cholesterol-lowering effect, with a 25% reduction of LDL-cholesterol and 35% of TG [17]; these effects were more evident in subjects that were not under therapy with other lipid-lowering drugs. The lipid-lowering effect of berberine was then evaluated in at least three meta-analyses [13,14,31]. The dose of berberine utilized in these studies was between 0.5 g and 1.5 g/day. The results clearly demonstrated that berberine reduces the LDL-cholesterol by approximately 25 mg/deciliter (dL) in patients with hypercholesterolemia and/or type 2 diabetes mellitus (T2DM). This variation was accompanied by a significant reduction in TG levels and a modest increase, albeit significant, of HDL-cholesterol levels [13,14,31].

Clinical evidence of the effect of berberine on circulating PCSK9 levels derives exclusively from studies conducted with combinations of nutraceuticals. For instance, the treatment of dyslipidemic subjects for 4 weeks with a nutraceutical formulation containing red yeast rice (monacolin K 3.3 mg), berberine 531.25 mg and leaf extract of *Morus alba* 200 mg, did not modify PCSK9 plasma levels [32]. The authors speculated that monacolin K, the statin produced by *Monascus purpureus* present in this combination, should increase plasma PCSK9 [33], and this effect may have been counteracted by the presence of berberine, a well-known negative modulator of PCSK9, as well as potentially by leaf extract of *Morus alba* [34]. Very similar results were observed in a double blind, randomized, placebo-controlled study that investigated the lipid-lowering effect of 12 weeks treatment with a nutraceutical containing chitosan, red yeast rice, and berberine, in individuals with hypercholesterolemia [35]. As expected, the treatment significantly reduced non-HDL-cholesterol and LDL-cholesterol compared to the placebo, while no changes were observed in PCSK9 plasma levels [35], further supporting the counteracting effect of berberine on monacolin K. On the contrary, the treatment of hypercholesterolemic patients with a nutraceutical combination of monacolin K, berberine, and silymarin determined a significant increase of PCSK9 plasma levels after 8 weeks [36]. This effect is likely due to the use of a different ratio of the monacolin and berberine doses, resulting in the increasing effect on PCSK9.

In an additional study, conducted in genotype-confirmed heterozygous familial hypercholesterolemic (HeFH) patients treated with statins or statins/ezetimibe combination, the supplementation with a nutraceutical containing berberine induced a further 10.5% reduction of plasma LDL-cholesterol level [37]. The mechanisms underlying this effect might consist of: (*i*) an increased expression of LDLR, encoded by the wild-type allele, coupled with their prolonged half-life; and/or (*ii*) a reduced expression of PCSK9. Unfortunately, in this study the levels of PCSK9 were not measured. However, the authors observed an inverse correlation between the reduction of LDL-cholesterol levels obtained with statins or statins/ezetimibe and the additional decrease induced by berberine [37]. Interestingly, a direct relationship between the hypolipidemic effect of statins and increased levels of PCSK9 has been observed [38,39], and the above-mentioned inverse correlation might be explained by the berberine-mediated inhibition of PCSK9.

Although berberine is usually very well tolerated at doses up to 1 mg per day, among its possible side effects are constipation, diarrhea, abdominal distension, and bitter taste. However, these effects were observed mainly in trials conducted with the highest doses [31]. It is also important to know that long-term administration of berberine was shown to reduce the activity of CYP2D6, CYP2D9 and CYP3A4 in healthy subjects [40], effects potentially associated with drug—drug interactions.

Another aspect needing further investigation is related to the bioavailability of different berberine preparations. Although it seems clear that berberine supplementation produces favorable effects on lipid metabolism, it is equally true that absorption of intestinal berberine is often minor and has a wide inter-individual variability [23,24]. This aspect could determine a high variability of the efficacy of the nutraceutical.

In this regard, several attempts have been pursued in order to improve the bioavailability of berberine, including the synthesis of non-natural derivatives [30,41], as well as drug delivery nanotechnology [42,43]. For instance, the synthetic derivative 8-hydroxy-dihydroberberine can produce similar lipid-lowering effects to berberine when only a quarter of the original dosage of berberine is administered, thus suggesting a better pharmacokinetic profile [30]. A second approach was based on the synthesis of a new series of indole-containing tetrahydroprotoberberine [41]. This study led to the identification of a new compound with potent inhibitory PCSK9 activity, that promoted LDL-cholesterol uptake in HepG2 cells and had an oral bioavailability of 21.9% [41]. This compound also showed a significant in vivo hypolipidemic potency in hamsters fed a high fat diet (0.5% cholesterol), when administered at the daily dose of 30 mg/kg [41].

To improve berberine bioavailability, Ochin and Garelnabi developed a new formulation consisting of the encapsulation of the compound within PLGA-PEG nanoparticles to negatively modulate PCSK9 [42]. Although this formulation was shown to be active in reducing PCSK9 expression in vitro, a direct comparison to berberine with in vivo evidence of a better oral bioavailability is still missing. Moreover, in vivo evidence of improved activity of berberine was recently reported with rational designed micelle (CTA-Mic) developed for an effective liver deposition of berberine. This new formulation has excellent in vivo lipid-lowering activity, although the authors did not provide data on PCSK9 levels [43].

## 3. Sterol/Stanols and Vegetable Proteins

Among available dietary supplements/substituents for cholesterol reduction, plant sterols/stanols have one of the widest uses [44]. There are, however, no clear data on these compounds, essentially showing no activity on PCSK9 levels and, in any case, they are not conclusive. Two groups have investigated the involvement of PCSK9 in the LDL-C lowering effect of plant stanols intake [45]. Simonen et al., in a randomized controlled double-blind trial in normal and hypercholesterolemic subjects, evaluated the effect of a 6 months consumption of vegetable-oil spread (20 g/day), enriched (plant stanol group) or not (control group) with plant stanols (3 g/day) as ester. The long-term intake of plant stanol esters reduced LDL-C by 7–10%, without affecting either PCSK9 plasma concentrations or the hepatic LDLR levels, indicating that plant stanol esters can lower LDL-C through inhibition of cholesterol absorption, without interfering with PCSK9 metabolism [46]. De Smet et al. showed that an acute intake of plant stanol esters (0.25 mg cholesterol + 50 mg plant stanol esters dissolved in olive oil) in mice up-regulated mRNA expression of intestinal PCSK9 and LDLR, and their main transcription factor SREBP-2, whereas hepatic expression of these genes was down-regulated after 15 min following oral intake. In parallel, reduced intestinal cholesterol absorption and decreased plasma LDL-C levels occurred [47].

Several food peptides from vegetable sources exert instead a cholesterol-lowering activity by a physical interaction with bile acid micelles [48]. These again show, however, no activity on PCSK9. A more interesting case is instead that of food peptides lowering LDL-cholesterol by statin-like mechanisms. This is the case of both soy and lupin peptide mixtures, achieving inhibition of HMG-CoA reductase activity of > 50% at 0.5 mg/mL levels [49]. A similar mechanism has been reported for soy β-conglycinin [50]. In addition, hempseed peptides (from *Cannabis sativa* L. Cannabaceae) appear to exert a hypocholesterolemic activity by a statin-like mechanism [51]. The mechanism of the HMG-CoA reductase inhibition was postulated for some peptides (TPMASD, HFKW and PMAS), based on molecular docking studies and enzyme assays, consistent with 3-dimensional similarity to statins [52]; the inhibitory activity was however far lower than the nanomolar IC_50_ values of known statins.

### 3.1. Lupin

**Figure 1:**
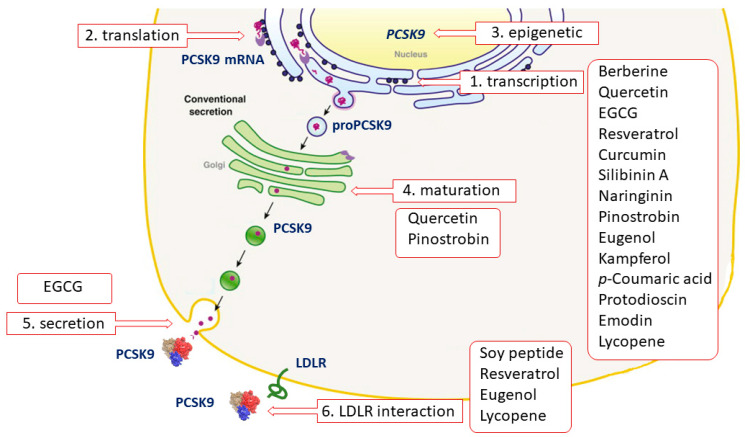
Schematic representation of the to-date-known mechanism of action of natural Proprotein convertase subtilisin/kexin type 9 (PCSK9) inhibitors.

Lupin is a protein-rich grain legume, is commonly represented by four domestic species, i.e., *Lupinus albus* (white lupin), *L. luteus* (yellow lupin), *L. mutabilis* (pearl lupin) and *L. angustifolius* (sweet leaf lupin; Fabaceae). Lupin proteins have been studied for a number of years mainly for their activity on plasma cholesterol reduction, attributable in large part to an LDLR-activating mechanism [53]. In animal models, lupin proteins have displayed hypolipidemic and a remarkable antiatherosclerotic effect [54]. Clinically, lupin proteins have been tested predominantly in hypercholesterolemic patients, with a positive effect on LDL-cholesterol and on the LDL:HDL cholesterol ratio shown in two studies, one with supplementation [55] and the other with diet enrichment [56]. Conversely, lupin protein combinations with cellulose led to a remarkable hypocholesterolemic effect [57], with a concomitant reduction of PCSK9 plasma levels (−8.5% vs. control) [58]. This trend was further confirmed in another randomized trial, on metabolic syndrome patients, in whom the dietary intervention with lupin proteins led to an 8% drop in LDL-cholesterol, with a decrease of 12.7% (vs. baseline) of PCSK9 levels [59]. Very recently, a mechanism has been hypothesized, i.e., in HepG2 cells, lupin proteins decrease both PCSK9 and HNF1α protein levels (Figure 1) [60]. In addition, lupin protein-derived peptides were found to inhibit the interaction between PCSK9 and LDLR, with the peptide LILPKHSDAD generated from lupin β-2 conglutin being the best candidate (Figure 1 and Table 1). This peptide dose-dependently inhibits PCSK9-LDLR binding, thus increasing LDL-uptake in HepG2 cells [61]. Besides inhibiting this interaction, these peptides have been found to lower the expression level of PCSK9 protein, thus reducing circulating enzyme levels. Thus, two main hypotheses could explain the activity of lupin proteins on PCSK9: (*i*) inhibition of protein–protein interaction between PCSK9 and the LDLR [61]; and (*ii*) reduced protein expression of HNF1α [58], at least in HepG2 cells (Figure 1).

This novel inhibitory pathway of functional foods, related to both the LDLR upregulation and possible PCSK9 antagonism, are of major interest these days, and may lead to new approaches to cardiovascular prevention.

### 3.2. Soy Proteins

Soy proteins are the most widely evaluated dietary proteins for metabolic control [62]. Proteins from *Glycine max* are the prototype plant proteins and, as such, have reached the attention, as reported in a recent position paper [63], on the effects of plant vs. animal protein sources on cholesterol reduction. The intake of active daily soy doses, in a range of 30 g, leads to an LDL-cholesterol reduction between 3% and 10%, an effect not associated with changes in PCSK9 circulating levels [64].

## 4. Polyphenols

Polyphenols are plant-derived secondary metabolites found in fruits, vegetables, nuts, seeds, herbs, spices, stems and flowers, as well as in tea and red wine. This class includes a huge number of different molecules such as flavonoids, lignans, stilbenes, and condensed (flavan-3-ol polymers known as proanthocyanidins) or hydrolyzable (such as tannic acid) phenolic polymers [65]. Several epidemiological studies, as well as clinical trials, have reported many cardiovascular benefits of polyphenols, occurring through multiple mechanisms of action, including plasma LDL-cholesterol-lowering activity [66,67,68]. From the mechanistic side, most of these molecules act by upregulating the LDLR at the hepatic surface, as described for berberine in the above section. This evidence led researchers to investigate the potential influence of polyphenols on PSCK9.

Although some data is available on the effect of polyphenols on PCSK9, it must be remembered that the main problem related to research on polyphenols in vitro is that concentrations of tested compounds are often higher than those detected in vivo, limiting the physiological relevance of these observations. In addition, the extensive metabolism that polyphenols undergo by intestinal microbiota in vivo may generate bioactive compounds, making it very hard to traverse the in vitro findings to the in vivo situation. We critically discussed these aspects that have been deeply reviewed elsewhere [69,70].

### 4.1. Quercetin

Quercetin [2-(3,4-dihydroxyphenyl)-3,5,7-trihydroxy-4*H*-chromen-4-one] is a flavonoid ubiquitous in fruit and vegetables (Table 1). Quercetin strongly upregulates the LDLR gene expression in hepatic cells, resulting in increased LDL uptake. This effect seems to be mediated by the activation of the transcription factor SREBP2 [71].

In vitro studies revealed that quercetin in its glicosidated form, incubated with HepG-2 cells at a concentration range from 1 to 10 µM, reduced PCSK9 mRNA levels by 20–30%. In addition, authors observed a 20–90% increase in intracellular PCSK9 levels and a 30–35% reduction in PCSK9 secretion in the culture medium [72]. The latter effect occurred through a negative modulation of sortilin, a protein inducing the cellular secretion of PCSK9 from the trans-Golgi network to the plasma membrane (Figure 1) [73]. Interestingly, quercetin 20 µM affects the expression of PCSK9 not only in hepatic cells, but also in a foam cell macrophages model [74]. This may unravel a direct antiatherogenic and LDL-cholesterol-independent effect of quercetin, since PCSK9 negatively modulates cholesterol metabolism and inflammation in macrophages [75,76]. Differently from hepatic cells and macrophages, quercetin 3-glucoside increased both PCSK9 and LDLR expression in mouse pancreatic cells. However, this was interpreted as a beneficial effect. In fact, the greater increase of PCSK9 relative to LDLR induced by quercetin may prevent cholesterol uptake, thus avoiding cholesterol-dependent dysfunction in these cells [77].

It should be noted that the high concentration of quercetin, and the use of its glicosidated form instead of the free aglycone, the active component, strongly limits the relevance of the above in vitro works. However, similar effects on PCSK9 were also observed in vivo, in which the enzymatic cleavage by the microbiota releases aglycon, which could undergo absorption. In fact, the supplementation with quercetin-3-gucoside (0.05 and 0.1% w/w) in high-cholesterol diet-fed mice reduced PCSK9 circulating levels, leading to increased LDLR expression at the hepatocyte surface. As observed in vitro, the supplementation significantly increased the amount of pancreatic PCSK9 [77]. A reduction of PCSK9 expression, both in liver and aorta, has also been observed after supplementation for 12 weeks with 12.5 mg/kg of quercetin, in apoE^−/−^ mice fed with a high-fat diet, suggesting a anti-atherogenic effect occurring at multiple levels (Figure 1 and Table 1) [78].

Specific clinical evidence of the effect of quercetin on circulating PCSK9 levels is still missing. However, several studies in humans undoubtedly highlighted the cholesterol-lowering properties of this flavonoid. As example, quercetin supplementation has shown to reduce by approximately 12% LDL-C levels, as emerged from a recent meta-analysis of randomized controlled trials [79].

From the pharmacokinetic side, like the most of polyphenols, quercetin is characterized by a poor solubility and low oral absorption, leading to physiological plasma concentrations lower than micromolar levels [80]. Moreover, quercetin is known to be a substrate and inhibitor of the P-gp and of breast cancer resistance protein (BCRP), and this further reduces its bioavailability (Table 1) [81,82,83,84]. Quercetin glycosides, the major form present in nature, undergo deglycosylation in the intestine, generating the quercetin-free form that successively is a substrate of liver enzymes [85], responsible for the production of the metabolites quercetin-3-sulfate, quercetin -3′-sulfate, and quercetin-3-glucuronide [86]. Quercetin is also metabolized by gut microbiota into 3,4-dihydroxyphenylacetic acid, 3-(3-hydroxyphenyl) propionic acid, 3,4-dihydroxybenzoic acid and 4-hydroxybenzoic acid [85].

Several formulations have been made to improve polyphenol bioavailability, by enhancing their solubility or preventing their degradation or metabolism [87]. Among them, a novel lecithin-based formulation of quercetin has been tested in healthy volunteers, showing a significant improvement in solubility, and consequently bioavailability [88].

### 4.2. Epigallocatechin Gallate

Epigallocatechin gallate (EGCG), the most active catechin found in green tea, has shown hypocholesterolemic activity occurring by an increase of LDLR mRNA levels and protein expression in human hepatoma cells line, in an ERK-signaling pathway-dependent manner. Moreover, EGCG reduced the production of apolipoprotein B (apoB), the main protein component of LDL [89]. This effect was shown to be independent of the 67 kDa laminin receptor, the main receptor described for EGCG [90]. Other evidence of the mechanism of action of EGCG is provided by Li and colleagues, who demonstrated EGCG’s capacity to inhibit the endogenous cholesterol synthesis via the suppression of SREBP2 with a sirtuin 1/forkhead box protein O1 (SIRT1/FOXO1) signaling pathway-dependent mechanism [91].

A marked reduced secretion of PCSK9 was observed in hepatic cells treated with 25 µM EGCG, with a maximum effect already evident after 3 h of incubation. In the same study, EGCG was able to counteract the inducing effect of lovastatin on PCSK9 secretion (Table 1). These effects were not accompanied by changes in PCSK9 mRNA, or in the intracellular precursor/mature protein level (Figure 1) [92].

Direct evidence of EGCG’s effect on circulating PCSK9 in humans is not yet available. However, several studies found a significant association between green tea drinking and lower plasma levels of total and LDL-cholesterol. For instance, the isolated EGCG has shown hypocholesterolemic effects (LDL-cholesterol −9.29%) in healthy subjects [93]. Similarly, the administration for 6 weeks of green tea extract lowered, by about 5%, LDL-cholesterol in overweight and obese women [94].

As discussed for quercetin, the oral bioavailability of EGCG is also low in humans (Table 1) [95]. The administration of 300 mg/day for 4 days, followed by an extra 150 mg the fifth day, led to a mean Cmax of 275.4 µg/mL, with more than sixfold variability among individuals. The reason for such variability is the high rate of metabolism: ECGC is mainly biotransformed in the liver and in the small intestine, leading to methylated, sulfated and glucuronidated metabolites, as well as phenylvalerolactones and phenylvaleric acids, that successively undergo glucuronidation [85,96,97]. The bioavailability of EGCG is also influenced by polymorphism in genes coding for multidrug resistance-associated protein 2 (MRP2) and organic anion transporter polypeptide 1 B1 (OATP1B1), transporters involved in the excretion and uptake of these molecules [98].

### 4.3. Resveratrol

Resveratrol (3,5,4′-trihydroxy-trans-stilbene) is a non-flavonoid polyphenol first isolated and identified from the roots of *Veratrum grandiflorum (Maxim. ex Miq), O. Loes (Melanthiaceae)*, and it is found in red wine, grapes, and peanuts (Table 1). It was previously demonstrated that red wine polyphenols upregulated LDLR expression and activity, and suppressed the secretion of apolipoprotein B-100 from human HepG2 cells. After this discovery, researchers specifically focused on the mechanism of action of resveratrol, the main bioactive polyphenol, finding a remarkable effect in inducing the transcription of the *LDLR* gene in hepatic cells, specifically occurring through the processing of SREBP, but independently of the adenosine monophosphate-activated protein (AMP) kinase (AMPK)-mediated signaling pathway [99]. Resveratrol also induced LDLR mRNA levels and protein expression in steatotic hepatic cells, by acting on the PCSK9 promoter with a mechanism involving SREBP1c [100]. In the same cells, 20 µM resveratrol reduced the expression of PCSK9 and promoted LDL uptake, with important implications for the pathogenesis of non-alcoholic fatty liver disease (NAFLD), the leading cause of liver damage [100]. The upregulating action on LDLR has been also seen for polydatin (piceid), the resveratrol natural precursor [101]. Indeed, polydatin has shown a potential interfering action on the PCSK9/LDLR interaction, as suggested by an in vitro screening work (Figure 1) [102]. The direct binding of polydatin to the active pocket of PCSK9 has been further highlighted, demonstrating that this interaction occurs through several hydrogen bonds. In the same study, authors found that the treatment with 20 µM of polydatin abrogated the inducing effect of palmitic acid on PCSK9 protein levels in an insulin-resistant hepatic cell model, suggesting a potential beneficial effect of polydatin on T2DM [101]. As discussed for quercetin, a strong limitation of these studies relates to the use of glucosides instead of free aglycone.

The beneficial effect of polydatin in the context of glucose intolerance and diabetes emerged also from in vivo studies: gene and protein expression of PCSK9 was found reduced in the liver and serum of diabetes (db/db) C57BL/6 mice treated with polydatin 100 mg/Kg, 6 d/week for 4 weeks. This effect was accompanied by an improvement in glucose metabolism, by a PCSK9-dependent upregulation of glucokinase (GCK) [101].

Concerning humans, no data are available so far on the effect of resveratrol on PCSK9, and even its efficacy on the lipid profile itself is still debated. The results of a recent metanalysis of 20 studies did not find an association between the administration of resveratrol and the LDL-cholesterol plasma levels, suggesting that the described cardioprotective effects of resveratrol may occur through an influence on other factors beyond lipids [103]. On the other hand, the results of another metanalysis concluded that longer resveratrol intervention trials (≥3 months) led to a significant reduction of plasma LDL-cholesterol [104]. Based on these available data, wider and longer studies are still needed to unequivocally determine the hypocholesterolemic effects of resveratrol.

Resveratrol demonstrates photosensitivity, poor solubility, and rapid metabolism, with negative consequences on bioavailability and bioactivity. The administration of an oral dose of 25 mg of resveratrol in humans resulted in plasma concentration from 1 to 5 nanograms (ng)/mL [105]. Due to its lipophilic nature, resveratrol may accumulate in several tissues and organs such as the brain, liver, and the intestine. About 20 resveratrol-derived metabolites have been reported in human plasma, urine, and human tissues. Among these, resveratrol-3-O-sulfate is reported as the most abundant liver-derived circulating metabolite [106]. Resveratrol and its metabolites may also be biotransformed in the colon by the gut microbiota, leading to generation of dihydroresveratrol [107].

### 4.4. Other Polyphenols

Few other polyphenolic compounds have demonstrated an influence on PCSK9 from preliminary data obtained in vivo or in vitro. However, data are too scarce and further investigations are needed to better characterize the bioactivity of these compounds with respect to PCSK9. For instance, silibinin A, a flavonolignan, has emerged as a repressor of PCSK9 promoter activity from the results of a drug-screening assay (Figure 1 and Table 1) [108]. In HepG2, increasing concentrations of silibinin A, from 10 to 100 µM, reduced PCSK9 mRNA levels and protein expression in a dose-dependent manner. This activity was dependent on the suppression of the p38 mitogen-activated protein kinase (MAPK) pathway. Importantly, silibinin A was able to attenuate the atorvastatin-induced PCSK9, with a complete counteracting effect observed at 50 µM, suggesting silibinin A as promising agent to abrogate the negative effect of statin on PCSK9 [108,109]. Silibin can be metabolized by both the liver, generating sulfate and glucuronide derivatives [109], and by the gut microbiota, leading to demethylated compounds, as highlighted by an ex vivo study [110].

Naringin, a flavanone-7-O-glycoside (naringenin 7-O-neohesperidoside), isolated from grapefruit and other citrus (Rutaceae), administered at doses of 25, 50 or 100 mg/kg/day for 8 weeks, reduced the hepatic expression of PCSK9, SREBP1 and SREBP2 in obese mice, and the LDLR was consequently induced (Figure 1 and Table 1). Plasma levels of PCSK9 and LDL-cholesterol were also measured and they have been found to both be dose-dependently reduced by naringenin [111]. When orally administrated, naringin is hydrolyzed to its aglycon naringenin by hydrolase and intestinal microflora [112]. Naringenin is partly absorbed and then engaged in both phase I and phase II metabolism. Meanwhile, unabsorbed naringenin and the metabolites excreted by the enterohepatic circulation are further degraded into phenolic catabolites by intestinal microbiota [112].

Finally, pinostrobin, a flavanone found in honey and in other plants [*Pinus strobus* L. Pinaceae, *Cajanus cajan* (L.) Millsp., Fabaceae, *Boesenbergia rotunda* (L.) Mansf., and *Boesenbergia pandurata* (Roxb.) Schltr., Zingiberaceae], was studied with respect of its potential influence on PCSK9. The treatment of HepG2 with 20 and 40 µM of pinostrobin led to a dose-dependent reduction of mRNA and protein expression of PCSK9, and a reduction of its catalytic activity, resulting in increased LDLR expression and LDL uptake by the cells [113]. Stereospecific differences in the pharmacokinetic profile of pinostrobin have been observed in rats after iv and oral administrations (Figure 1 and Table 1) [114].

### 4.5. Eugenol

Eugenol (4-allyl-2-methoxyphenol), a major component of the essential oil of clove [*Syzygium aromaticum* (*L.*)], is a phenolic nutraceutical with known hypocholesterolemic activities (Table 1). It has been considered as a safe nutrient, with the acceptable daily intake of up to 2.5 mg/kg body weight in humans. Animal studies have shown that eugenol lowers serum cholesterol levels and inhibits lipogenesis in the liver, thus suggesting a protective effect on atherosclerosis and fatty liver disease [115,116]. More recently, a molecular docking analysis revealed hydrophobic interactions between ligand eugenol and PCSK9 (Figure 1) [117]. In addition, eugenol was found to reduce the expression of PCSK9 in Jurkat cells [117]. This effect can be the result of a physical interaction between the two molecules, or an indirect inhibitory effect of eugenol on the SREBP pathway (Figure 1) [115]. The pharmacokinetic profile of eugenol has only been investigated in experimental models (Table 1) [118].

## 5. Nutrients

### 5.1. Curcumin

Curcumin [1,7-bis(4-hydroxy-3-methoxyphenyl)-1,6-heptadiene-3,5-dione] is one of the main bioactive polyphenolic components of the spice turmeric, prepared from the rhizome of *Curcuma longa* L. (Zingiberaceae) (Table 1).

Curcumin increased the expression of the LDLR and LDL uptake in HepG2 in a dose- and time-dependent manner. This activity occurred through the activation of the SREBP pathway [119], although this result was not confirmed in other studies [120,121]. More recently, the stimulating effect of curcumin on LDLR expression and activity was further observed, but this increase was not accompanied by changes in *LDLR* transcription and mRNA stability, suggesting a regulation at the transcriptional level [122]. Indeed curcumin 10 and 20 µM for 24 h markedly reduced PCSK9 mRNA and protein expression in hepatic cells. In this work, the transcription factor HNF1α, but not SREBP, was involved in the curcumin-mediated effect on PCSK9. Interestingly, curcumin almost completely abrogated the PCSK9-inducing effect of lovastatin, suggesting that curcumin could counteract the effect of statin on circulating PCSK9 [123] and opening new perspectives on novel nutraceutical cholesterol-lowering combination approaches (Figure 1). However, the higher concentrations of curcumin, although widely used in cell culture system, are higher than what can be achieved in vivo, reducing the relevance of these findings.

The only evidence of curcumin influence on PCSK9 in vivo was reported in 2017. The authors suggest an anti-endotoxemic action of curcumin, that would be able to improve LPS detoxification via the LDLR. In detail, authors observed that treatment of cirrhotic rats with curcumin 200 mg/kg/day for 12 weeks, despite no change in mRNA, induced an increase in LDLR protein expression in their liver. This occurred because of a curcumin-inhibition effect on PCSK9 mRNA and protein level [124].

Although no report is so far available on the influence of curcumin on PCSK9 in humans, several studies have examined the effect on LDL-cholesterol levels. Results of these investigations are controversial [125], reporting weak effect or no change, as suggested by the results of a metanalysis of randomized controlled trials [126]. The reason for this discrepancy may be related to the population studied, to the length of treatment and the type of formulation that can lead to different bioavailability.

Indeed, low poor aqueous solubility, bioavailability, and an unfavorable pharmacokinetic profile, limits curcumin’s therapeutic use. In particular, curcumin presents a poor stability under physiological conditions, with a t_1/2_ of less than 10 min [127]. Curcumin and its hepatic-derived metabolites, mainly conjugated with glucuronide, sulfate and glutathione, are further transformed by the gut microbiota, generating more than 10 different molecules, including tetrahydrocurcumin, demethylcurcumin, bisdemethylcurcumin, etc. [128,129]. To overcame curcumin pharmacokinetic issues, several formulation approaches have been proposed, that will need to be tested in appropriate pharmacological studies [130].

### 5.2. Welsh Onion

Welsh onion (*Allium fistulosum L*., Amaryllidaceae) is a perennial plant that is widely cultivated throughout the world, especially in Asia. The ethanol extract of welsh onion contains 0.5 g/100 g of total fat, and is rich in vitamins B2 (riboflavin, 1.3 mg/100 g), B3 (niacin, 284.3 mg/100 g), B6 (pyridoxine, 5.4 mg/100 g), and B9 (folic acid, 2.2 mg/100 g), and mineral iron (20.8 mg/100 g). In addition, welsh onion contains 0.53 ± 0.02 mg/g of ferulic acid and 0.61 ± 0.01 mg/g of quercetin [131]. A second study confirmed that ferulic acid is the most abundant phenolic compound present in welsh onion extract (0.16 ± 0.01 mg/g of extract), followed by *p*-coumaric acid and kaempferol (0.11 ± 0.01 and 0.10 ± 0.01 mg/g of extract, respectively) [132]. Quercetin was also found in the extract, but the amount was negligible (0.04 ± 0.01 mg/g extract).

In HepG2 cells, welsh onion ethanol extract was shown to control the induction of different genes involved in lipid and cholesterol metabolism in response to lipid-deprived serum [132]. The extract was active at 50 μg/mL up to 200 μg/mL concentration, and effectively controlled LDLR protein expression. Importantly, at the same concentrations, a significant reduction of PCSK9 mRNA levels were also observed [132], suggesting a negative impact on gene transcription. In accordance with this hypothesis, the authors observed a strong reduction of both SREBP2 and HNF1α [132]. Welsh onion ethanol extract also reduced PCSK9 protein expression, determined by western blot analysis of total protein extracts, without any significant changes in the LDLR levels. These data suggest that, despite a significant inhibitory effect on PCSK9, welsh onion did not increase LDLR expression [132].

The ethanol extract was also shown to counteract the induction of PCSK9 by statins, further supporting a negative effect on SREBP or HNF1α-dependent regulation of PCSK9 transcription [132].

Among the active components identified in the extract, kaempferol, quercetin, and *p*-coumaric acid significantly reduced the PCSK9 level under lipid depletion conditions in HepG2 cells, albeit this effect was observed at considerably high in vitro concentrations (40 μM). On the contrary, ferulic acid did not show any significant effect (Figure 1 and Table 1) [132].

The hypolipidemic effect of welsh onion ethanol extract was investigated in C57BL6/J mice fed a high-fat diet (60% of energy as fat, 20% as protein and 20% as carbohydrates) [133]. Welsh onion extract was dissolved in normal saline and was orally administered to the mice at a dose of 400 mg/kg/day for 6.5 weeks. This supplementation lead to a significant reduction of body weight and food intake, with a significant reduction of TG (−46%), TC (−11%) and LDL-cholesterol (−24%) [133]. Interestingly, the authors also observed a reduction in the expression of SREBP1c in the liver, confirming the data obtained in vitro [133] and suggesting a possible effect on PCSK9, although this analysis has not been performed [132].

### 5.3. Cashew Nuts (Anacardium Occidentale L., Anacardiaceae)

The last guidelines of European Atherosclerosis Society (EAS) and European Society of Cardiology (ESC) clearly state that higher consumption of fruit, non-starchy vegetables, nuts, legumes, fish, vegetable oils, yoghurt and wholegrains, along with a lower intake of red and processed meats, foods higher in refined carbohydrates, and salt, is associated with a lower incidence of cardiovascular (CV) events [134]. These data indicate that the replacement of animal fats with vegetable sources of fats and polyunsaturated fatty acids (PUFAs) may decrease the risk of CV disease (CVD). However, clinical trials relating cashew nuts to cardiovascular disease risk factors, including LDL-cholesterol, are limited to four conflicting studies [135,136,137,138]. In one controlled-feeding study conducted on a total of 42 adults as a randomized crossover trial, the addition of 42 g of cashews/day was associated with a significant reduction of PCSK9 plasma levels (270.8 ng/mL vs. 252.6 ng/mL) [135]. This effect was not associated to any significant change of the LDL-cholesterol, and the active component responsible for the inhibition of PCSK9 is still unknown.

### 5.4. Kenaf

Kenaf (*Hibiscus cannabinus* L., Malvaceae) and defatted kenaf seed meal (DKSM) is a low-cost agricultural waste, but potentially a value-added functional food ingredient with hypocholesterolemic properties [139]. Phenolics and saponins are two major bioactive classes in DKSM that confer superior antioxidant properties compared to common edible flours, i.e., wheat, rice and sweet potato flours [139]. The hypocholesterolemic effect of DKSM was recently tested in rats fed high-fat and cholesterol-containing atherogenic diet, containing either 15% or 30% DKSM. Alternatively, rats were fed with the same diet but supplemented with 2.3% or 4.6% of phenolic-saponin rich extract (PSRE) of DKSM. The main active components detected in DKSM or PSRE were *p*-coumaric acid, caffeic acid, (+)-catechin and gallic acid [139].

Supplementation with DKSM, and the equivalent levels of PSRE, in hypercholesteremic rats for 10 weeks, exhibited substantial atherogenic risk reduction, with reduced levels of total and LDL-cholesterol and increased HDL-cholesterol [139]. DKSM and PSRE reduced HMG-CoA reductase in the liver, and more importantly serum PCSK9 levels. These effects are probably to be attributed to phenolic and saponin components. *p*-Coumaric acid, caffeic acid, (+)-catechin and gallic acid have been reported to exhibit anti-hypercholesterolemic properties in different animal models [140,141,142]. In particular, saponins appear to interfere with SREBP transcription factor, and are the most likely components that affected PCSK9 expression (Figure 1 and Table 1) [143].

### 5.5. Vitamin MK7

Vitamin K occurs in two dietary forms, i.e., vitamin K1 (phylloquinone) and vitamin K2 (menaquinones, MK). Vitamin K2 is mainly found in fermented foods such as cheese and “natto”, a Japanese soybean product [144]. More than 12 different types of MK-n have been identified, from MK-4 to MK-15, where “n” indicates the number of unsaturated isoprenoid residues linked to the menaquinone (Table 1). MK-7 is produced mainly by submerged fermentation using *Bacillus subtilis* and shows a more favorable pharmacokinetic profile compared to MK-4, including a longer half-life time and higher bioavailability [145]. After its intestinal absorption, vitamin K is solubilized by bile salts and pancreatic juice and packaged into chylomicrons [146]. European experts suggested that the Recommended Daily Intake (RDI) of vitamin K, preferably in the form of vitamin K2, is 200–500 μg/day (200 µg/day for MK7), which is required for optimal carboxylation of extrahepatic γ-carboxyglutamic acid (GLA)-proteins.

The hypocholesterolemic action of vitamin K derives from an old study conducted on chronic renal failure patients treated with continuous ambulatory peritoneal dialysis. Vitamin K2 was administered at very high dose (45 mg daily) for several months, and the biochemical analysis showed that TC concentrations at 3 months were significantly higher than those at 7 months or later. Similar effects were observed on LDL-cholesterol [147].

More recently, we have observed a reduction of TC levels in uremic rats after the administration of a nutraceutical combination named RenaTris^®^, containing MK-7, magnesium carbonate, and Sucrosomial^®^ Iron [148]. By in vitro experiments conducted in hepatoma cells, it was found that MK7 alone reduces the cholesterol biosynthesis, by potentially affecting an enzymatic step of the mevalonate pathway upstream of the squalene synthase [148]. In response to the inhibition of cholesterol synthesis, MK7 induces LDLR, similarly to statins, and this effect was prevented by the co-incubation with squalene [148]. However, differently from statin, which induces PCSK9 expression, MK7 was shown to suppress PCSK9 synthesis and secretion by hepatoma cells [148]. This is thus very similar to what is observed with berberine, although the mechanism of action of MK7 is still unknown (Table 1).

### 5.6. Lycopene

Lycopene belongs to the family of the lipid-soluble antioxidants called carotenoids, which are found in fruits and vegetables [149,150] but mainly present in tomatoes, or tomato-containing products, which account for about 80% of total lycopene ingestion (Table 1). Growing evidence points to several beneficial effects of lycopene in the maintenance of CV function and health. Among the carotenoids, lycopene exhibits the highest potent antioxidant activity, but it seems to exert additional cardioprotective functions, such as anti-inflammatory properties, platelet aggregation inhibition and endothelial protection [151]. In a recent in vivo study, it was shown for the first time that lycopene administration in hypertriglyceridemic rats (5, 10 and 50 mg/kg body weigth/day) suppressed the hepatic PCSK9 mRNA expression two- and threefold through the ubiquitin-induced proteasomal degradation of HNF1α [152]. This effect partly explains the reduced plasma levels of atherogenic lipoproteins in lycopene-treated rats, as treatment with lycopene significantly decreased the level of plasma LDL-cholesterol and very low-density lipoprotein (VLDL)-cholesterol, as well as TG, with the maximum effect reached at the highest dose (−85.3%, −55.5% and 55.5%, respectively). In light of the reported reciprocal regulation between PCSK9 and inflammatory cytokines [153], the authors [152] hypothesized that the lycopene-induced inhibition of PCSK9 expression in hypercholesterolemic rats might be related to the suppression of inflammatory markers mediated by lycopene, as treatment induced a significant decrease, of 45, 39.3%, 29.8% and 47.8%, respectively, in the concentrations of circulating interleukin (IL)-1β, IL-6, tumor necrosis factor (TNF)-α and C-reactive protein (CRP), with maximum effect at the highest dose of lycopene. Finally, from in-silico molecular modelling studies, the authors demonstrated that lycopene reduces the affinity of PCSK9 with the complex EGFA (epidimal growth factor-A) of LDLR (Figure 1). In another study, the same group also showed that lycopene, through inhibition of HNF1α expression and possibly through the upregulation of farnesoid X receptor (FXR) and/or PPARα, reduces twofold the LPS-induced hepatic upregulation of PCSK9 in rats [154]. Again, the observed restoration of the inflammatory cascades in LPS-treated mice by lycopene treatment (−64.1%, −25.7%, 20% and −27.4% on circulating TNF-α, IL-1β, IL-6 and CRP, respectively, when compared to the LPS control group) is likely related to the suppression of PCSK9 expression (Figure 1).

The main problem is related to lycopene’s low bioavailability; in the human organism only 10–30% of the lycopene in trans-isomeric form is absorbed from the alimentary sources [155]. Its bioavailability depends on several factors, such as the different lycopene biochemical isoforms, the lycopene sources, doses, food co-ingestion, and genetic factors. Indeed, lycopene bioavailability and metabolism is strongly influenced by genetic variability, being described as at least 28 single nucleotide polymorphisms in 16 genes, among which are those coding for the cholesterol membrane transporter scavenger receptor class B, member 1 (SCARB1), the molecular guidance cue slit homolog 3 gene (SLIT3), and the steroid-breakdown enzyme dehydrogenase/reductase (SDR family) member 2 (DHRS2). New technologies to overcome bioavailability problems have been recently investigated, by testing nanodrugs in a nano-emulsion composed of lycopene as anti-inflammatory agent in an animal model of rheumatoid arthritis [156]. With respect to lycopene metabolites, it has been reported that the enzyme b,b-carotene 9′,10′-dioxygenase (BCO2) may catalyze the eccentric cleavage of both provitamin and non–provitamin A carotenoids to form apo-10′-carotenoids, including apo-10′-lycopenoids from lycopene [157], which have been demonstrated to mediate some of the biological activities of lycopene [158].

### 5.7. Omega 3

Omega-3 (ω-3 or n-3) polyunsaturated fatty acids (PUFA) are characterized by having the last double bond between carbon numbers 3 and 4 in the hydrocarbon (acyl) chain, counting the terminal methyl carbon as number one. Longer chain n-3 fatty acids include eicosapentaenoic acid (EPA; 20:5n-3), docosapentaenoic acid (DPA; 22:5n-3) and docosahexaenoic acid (DHA; 22:6n-3), found in significant amounts in fatty fish, fish oil and in other seafood. These exert a number of cardioprotective effects by favorably modulating several risk factors for CVD, such as blood lipids, blood pressure, heart rate and heart rate variability, platelet aggregation, endothelial function and inflammation [159]. With respect to cholesterol metabolism, EPA and DHA have been shown to reduce production, and may induce a faster clearance of triglyceride-rich lipoproteins (TGRL), with a paralleled more rapid clearance of LDL particles and slower production of VLDL particles [160]. These effects seem to be mediated by the inhibition of the SREBP1 mediated pathways, including the activation of the nuclear transcription factors, hepatocyte nuclear factor-4 alpha (HNF4), FXR, liver X receptor (LXR), and PPARs [161,162]. Studies conducted in animal models showed that long term intake of n-3 PUFA-enriched fish oil (10% in diet) reduces hepatic PCSK9 expression, with a consequent significant 84% reduction of LDL-cholesterol plasma levels [163], and that an omega-3 fatty acid-rich diet reduced PCSK9 plasma levels in association with 40% less plasma VLDL- and LDL-cholesterol [164]. Consistently, in subjects with at least one of the metabolic syndrome risk factors, a diet supplemented with canola oil enriched with DHA (by 6%) lowered circulating PCSK9 and TG levels compared to canola and canola oleic diets. In the same study, circulating PCSK9 levels were found to be significantly and positively associated with LDL-cholesterol, TG and apoB levels [165]. Moreover, daily consumption of marine n-3 PUFAs (containing 38.5% EPA, 25.9% DHA and 6.0% DPA) decreased circulating PCSK9 levels by 11.4% and 9.8% in premenopausal and postmenopausal women, respectively, without affecting plasma LDL-cholesterol levels [166].

Despite the several beneficial effects of long-chain omega-3 PUFA supplementation, DHA and EPA have been shown to also increase LDL-cholesterol levels [167], with DHA being more potent than EPA [168]. Consistently, in a recent study in men and women at high risk of cardiovascular disease, it has been observed that, compared with EPA, supplementation with DHA increased LDL-cholesterol concentrations (+3.3%; *p* = 0.038) and the mean LDL particle size, and reduced the proportion of small LDL (23.2%; *p* = 0.01) [169]. Despite the increase in LDL-cholesterol, compared to control both DHA and EPA reduced PCSK9 concentrations in a similar manner (DHA, −225.0 ng/mL; EPA, −218.2 ng/mL). Moreover, changes in PCSK9 correlated positively with changes in the LDL apoB-100 concentrations, and negatively with changes in LDL apoB-100 fractional catabolic rate, after DHA but not after EPA, suggesting a partial role of PCSK9 in the differential effects of DHA and EPA supplementation on LDL metabolism. Allaire et al. also observed that the responders to DHA or to EPA, in terms of TG reduction, had greater serum PCSK9 concentration at baseline than non-responders, suggesting a modulatory role of this protein in the n-3 PUFA-mediated effects [170]. With respect to the mechanism underlying the relationship between omega-3 and PCSK9, it has been hypothesized to be a modulation of SREBP2-mediated pathways [171]. In the context of the reciprocal regulating relation between long chain n-3 PUFAs and PCSK9, there has recently observed a significant interaction between the common *PCSK9* variant rs11206510 located in the promoter region of the *PCSK9* gene, identified for early onset myocardial infarction (MI) through a genome-wide association study (GWAS) [172], and long chain n-3 PUFA intake in Costa Rican Hispanics. Carriers of this variant reported a lower risk of nonfatal MI as compared to non–carriers [173].

Several omega-3 formulations naturally concentrated or purified from fish oil have been approved by the US Food and Drug Administration (FDA) for the treatment of severe hypertriglyceridemia. Some of these formulations provide EPA and/or DHA in either ethyl ester (EE), that requires digestion with carboxyl ester lipase (bile salt-dependent lipase). Therefore, the bioavailability of EPA and DHA from n-3 EE products is strictly dependent on their consumption with a fat meal to stimulate the release of bile salts. In this regard, technologies have been developed to enhance EPA and DHA absorption and to facilitate bioavailability [174]. A very recent study in humans showed that pre-digested omega-3-sn-1(3)-monoacylglycerol lipid structure (OM3-MAG) has a significantly greater absorption at high therapeutic doses (2.9 g/day) than the most common omega-3-EE (3.1 g/day) forms used in hypertriglyceridemia, suggesting the use of the pre-digested OM3-MAG as a more efficacious therapy in severe CV conditions, where high doses of omega-3 are required and a low-fat diet is indicated [175].

## 6. Other Inhibitors

### 6.1. Probiotics

Gut microbiota has a relevant impact on cholesterol metabolism, and thus on the pathogenesis of atherosclerosis [176]. For this reason, the use of selected probiotics with specific biological properties has been proposed as a new therapeutic approach for controlling hypercholesterolemia. Within this context, only one study reported data on PCSK9 levels [177]. This clinical trial evaluated the efficacy and safety of a nutraceutical combination containing *Bifidobacterium longum* BB536, red yeast rice extract, niacin and coenzyme Q10, on the improvement of LDL-cholesterol level, as well as the efficacy and safety of a set of clinical and experimental markers of cardiovascular risk. The results of this randomized, double-blind, placebo-controlled study demonstrated that 12 week-treatment significantly reduced TC (−16.7%), LDL-cholesterol (−25.7%) and apoB (−17%), without any changes in PCSK9 plasma levels. From the analysis of the circulating levels of lathosterol, markers of cholesterol synthesis, and campesterol, markers of intestinal cholesterol absorption, it was concluded that *Bifidobacterium longum* BB536 may counteract increased cholesterol absorption potentially induced by monacolin K present in the red yeast rice. On the same line, *Bifidobacterium longum* BB536 might dampen the induction of PCSK9 plasma levels observed in statin-treated patients. However, how *Bifidobacterium longum* BB536 may regulate PCSK9 expression is not known.

### 6.2. Dioscorea

The aqueous extracts from the root of the *Dioscorea zingiberensis* C.H. Wright, and from rhizome of *Dioscorea nipponica* Makino (Dioscoreaceae), have been used in the prevention and treatment of atherosclerotic CVD for nearly 30 years in China. In 2012, these products were also approved in the Netherlands. Several clinical reports have shown that *Dioscorea nipponica* can decrease the levels of TC, LDL-cholesterol and TG [178,179]. More recently, in a classical mouse model of atherosclerosis, involving apoE^−/−^ mice fed a high-fat diet for 18 weeks, dioscorea showed potent lipid-lowering and anti-atherosclerotic effects [179]. More importantly, dioscorea downregulated hepatic PCSK9 mRNA and reduced circulating PCSK9. The analysis of the composition of the extract of *Dioscorea nipponica* rhizome revealed the presence of protodioscin, pseudoprodioscin and dioscin. These steroidal saponins are considered the main active components. However, some dioscin terpenoids are conjugated with a polysaccharide and cannot be absorbed at gastrointestinal level, while their respective aglycones may be bioavailable. Interestingly, protodioscin, pseudoprotodioscin and methylprotodioscin have been shown to suppress PCSK9 expression (Figure 1 and Table 1) [180]. This effect was associated with the inhibition of SREBP transcription factors and was responsible of the induction of the LDLR protein in HepG2 cells [180]. It is still unclear whether the aglycone of protodioscin and pseudoprotodioscin is released under in vitro conditions, and thus the active component on PCSK9 is unknown. Indeed, HepG2 cells are known to show extremely low activity of numerous xenobiotic metabolizing enzymes, which could provide misleading results in pharmacological tests with compounds that require biotransformation [181,182,183]. This is particularly true for natural compounds that need to be activated by enzymes of gut microbiota that are not present in cultured cells.

### 6.3. Emodin

Emodin (6-methyl-1,3,8-trihydroxyanthraquinone) is one of the active anthraquinone derivatives from *Rheum palmatum* L. (Polygonaceae) and some other Chinese herbs (Table 1) [184]. In C57BL6/J mice fed high-fat diets for 12 weeks, emodin supplementation at the dose of 40 and 80 mg/kg/day showed an improvement of lipid levels associated with a reduction of SREBP expression [185]. In addition, in rats fed high-fat diets, emodin was shown to prevent hypercholesterolemic status through the bile acids-CYP7A1 pathway. Emodin binds and reduces the reabsorption of bile acids, leading to cholesterol being shunted into bile acid production, which determines its lipid-lowering effects [186]. More recently, 100 mg/kg per day of aloe, which also contains emodin, was shown to reduce TC and LDL-cholesterol levels in diet-induced hypercholesterolemic rats. Interestingly, aloe ameliorates the liver fat content, and in vitro studies on HepG2 cells show a negative effect on SREBP and HNF1α. As expected, the inhibition of both transcription factors determined a downregulation of PCSK9, associated with increased expression of LDLR and LDL uptake (Figure 1) [187].

Most of the individualized compounds have been shown to inhibit PCSK9 transcription factors, such as SREBP and HNF1α. However, there is evidence that compounds with different mechanisms of PCSK9 inhibition also exist, including: Epigallocatechin gallate (EGCG), which affects PCSK9 secretion; soy peptides, resveratrol, eugenol and lycopene, which inhibit the interaction of PCSK9 with the LDL receptor (LDLR); and finally, quercetin and pinostrobin, which impair the autocatalytic processing and maturation of PCSK9 in the endoplasmic reticulum. Today there is no evidence of natural compounds affecting PCSK9 at the translational level and by epigenetic mechanisms.

## 7. Conclusions

The relevance of PCSK9 as a new molecular target for treating hypercholesterolemia and associated cardiovascular diseases is demonstrated by the clinical efficacy of two FDA/EMA-approved monoclonal antibodies: alirocumab and evolocumab [5]. However, these monoclonal antibodies, the only currently available anti-PCSK9 therapies, have several drawbacks: (*i*) very high costs; (*ii*) subcutaneous administration (poor compliance and convenience); (*iii*) potential immunogenicity with long term treatment. A more recent alternative to anti-PCSK9 antibodies is represented by Inclisiran, a short interfering RNA (siRNA) designed to target hepatic PCSK9 mRNA. However, this approach still has some drawbacks, such as the long pharmacokinetic profile, parenteral administration, and an as yet undefined safety profile [193]. Thus, cheaper, orally administrable, small-molecule drugs are greatly needed. The response to this issue can potentially come from the identification of natural compounds with lipid-lowering activity associated with anti-PCSK9 inhibitory action. In the present review, we identified many compounds with effective anti-PCSK9 inhibitory activity, mainly by acting at the transcriptional levels, and only few examples of the autocatalytic secretion step or PCSK9 interaction with the LDL receptor. A critical aspect of all these potentially valid PCSK9 inhibitors is represented by their limited oral bioavailability, and the restricted evidence of their efficacy only in in vivo experimental models. Nevertheless, a number of drug delivery approaches and chemical derivatives of natural compounds, aiming to improve the oral bioavailability, are emerging. In addition, it must be recognized that, as elsewhere reviewed [194], when assessing the efficacy of a nutraceutical, the following key aspects are worth of consideration: (*i*) to identify differences in purity and origin between products on the market [195]; (*ii*) to find evidence of clinical efficacy evaluated via placebo-controlled, double-blind studies [196]; (*iii*) to evaluate the effects of combining active ingredients. Finally, evidence has to be grounded on the mechanisms of action of active ingredients in vitro, followed by pre-clinical studies in experimental animals, to finally explore safety and efficacy in humans [194]. All these aspects have not always been provided for the natural compounds described in the present review. Thus, the selected molecules can only be considered as starting points for the eventual development of oral PCSK9 inhibitors.

## Figures and Tables

**Table 1:** Pharmacokinetic and pharmacodinamic characteristics of natural compounds affecting PCSK9.

Natural Compound	Chemical Structure	Bioavail.	Tmax (h)	Half-Life Time (h)	Metabolism	Mechanism of Action	Transport.	Level of Evidence on PCSK9	Counteracts Statins	Ref.
Berberine	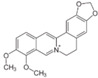	0.37%	9.8	28.6	Demethylation and glucuronide	Inhibits SREBP; HNF1α	P-gp and MRP1	In vitro, in vivo and clinical	Yes	[23,24,28,30,33,35,36,37,188]
**Sterol/Stanols and Vegetable Proteins**										
Lupin peptide	LILPKHSDAD	Poor (predicted)	Not known	Not known	Proteases	Inhibits interaction PCSK9-LDLR; Reduces HNF1α	No (predicted)	In vitro. Clinic (negative)	Not known	[61,64]
**Polyphenols**										
Quercetin	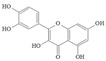	0.31%	2.5 ÷ 3	2.1	Liver: Sulfate and methyl glucuronide; Gut microbiota: free aglycone; 3,4-dihydroxyphenylacetic acid, 3-(3-hydroxyphenyl) propionic acid, 3,4-dihydroxybenzoic acid and 4-hydroxybenzoic acid	Inhibits secretion (sortilin) and SREBP	P-gp sand BCRP	In vitro and in vivo	Not known	[72,73] [78,80,81]
Epigallocatechin gallate	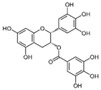	0.1%	1 ÷ 2	3.4	Liver: Methyl, sulfate, and glucuronide; Gut microbiota: phenylvalerolactones and phenylvaleric acids	Inhibits secretion and SREBP	MRP2 and OATP1B1	In vitro	Yes	[85,91,92,95,96,97,98]
Resveratrol	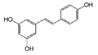	<1%	3	9.2	Liver: Sulfate and glucuronide; Gut microbiota: dihydroresveratrol	Inhibits SREBP1c and interaction PCSK9-LDLR	Not known	In vivo and in vivo	Not known	[100,101,102,105,106,107]
Curcumin	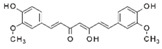	<1%	Not known	Not known	Liver: reduction and glucuronide, sulfate, and glutathione; Gut microbiota: tetrahydrocurcumin, demethylcurcumin, bisdemethylcurcumin etc.	Inhibits; HNF1α	Not known	In vitro and in vivo	Yes	[122,123,124,127,128,129]
Silibinin A	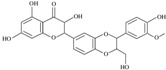	<1%	1.4	Not known	Sulfate and glucuronide	Inhibits transcription	P-gp inhibitor	In vitro	Yes	[108,109,110]
Naringin	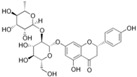	<1%	0.5	9.5	Liver: Hydrolysis and then glucuronide, sulfate, methylation; Gut microbiota: Phenolic derivatives	Inhibits SREBP	P-gp and OATP1A5 inhibitor	In vivo	Not known	[111,112]
Pinostrobin	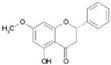	1.8% (*S*); 13.8% (*R*)	6.0	38.1	Glucuronide	Inhibits transcription and catalytic activity	Not known	In vitro	Not known	[113,114]
Eugenol	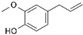	<1%	2.1	14.0	Phenol, glucuronide and sulphate	Direct interaction with PCSK9 and inhibits SREBP	Not known	In vitro	Not known	[115,117,118]
**Nutrients**										
Kaempferol	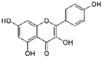	2.5%	Not known	Not known	Glucuronide and sulphate	Inhibits transcription	Not known	In vitro	Yes	[86,132]
*p*-Coumaric acid	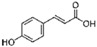	24%	0.17	0.25	Glucuronide and sulphate	Inhibits transcription	Not known	In vitro	Yes	[132]
Vitamin K7	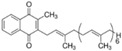	2%	6.0	60	Not known	Not known	Not known	In vitro and in vivo	Not known	[145,148,189]
Lycopene	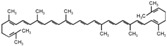	33.9%	24	235	Phase I, oxidation	Inhibits transcription and interaction PCSK9-LDLR	Not Known	In vitro and in vivo	Not Known	[151,152,154,190]
**Other Inhibitors**										
Protodioscin	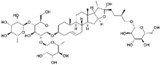	0.2%	20	20	Oxidation, deglycosylation and glucuronide	Inhibits transcription	Not known	In vitro and in vivo	Not known	[179,180]
Emodin	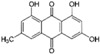	low	0.13	8.6	Glucuronide and sulphate	Inhibits SREBP; HNF1α	Not known	In vitro	Not known	[191,192]
